# Supplementary material for: Rapid and Reproducible MALDI-TOF-Based Method for the Detection of Vancomycin-Resistant Enterococcus faecium Using Classifying Algorithms
Source: Diagnostics (Basel). 2022 Jan 27;12(2):328. doi: 10.3390/diagnostics12020328 (PMC8871047; doi:10.3390/diagnostics12020328)

Table S1. Description of the 178 *E. faecium* isolates included in this study.

	Strain ID	Source	Resistance mechanism
**1**	2-17	Blood culture	vanA
**2**	2690-17	Rectal swab	vanA
**3**	2691-17	Rectal swab	vanA
**4**	2799-17	Rectal swab	vanA
**5**	2800-17	Rectal swab	vanA
**6**	1281-17	Rectal swab	vanA
**7**	250-18	Rectal swab	vanA
**8**	1290-18	Rectal swab	vanA
**9**	1552-18	Rectal swab	vanA
**10**	1785-18	Rectal swab	vanA
**11**	2051-18	Rectal swab	vanA
**12**	2082-18	Rectal swab	vanA
**13**	2271-18	Rectal swab	vanA
**14**	2272-18	Rectal swab	vanA
**15**	3307-18	Rectal swab	vanA
**16**	3412-18	Rectal swab	vanA
**17**	3549-18	Rectal swab	vanA
**18**	31-19	Rectal swab	vanA
**19**	40-19	Rectal swab	vanA
**20**	87-19	Rectal swab	vanA
**21**	92-19	Rectal swab	vanA
**22**	262-19	Rectal swab	vanA
**23**	457-19	Rectal swab	vanA
**24**	942-19	Blood culture	vanA
**25**	947-19	Blood culture	vanA
**26**	968-19	Rectal swab	vanA
**27**	1355-19	Rectal swab	vanA
**28**	1538-19	Rectal swab	vanA
**29**	1756-19	Rectal swab	vanA
**30**	19138416-19	Rectal swab	vanA
**31**	3024-19	Rectal swab	vanA
**32**	313-17	Blood culture	vanB
**33**	342-17	Blood culture	vanB
**34**	2038-17	Blood culture	vanB
**35**	107-18	Rectal swab	vanB
**36**	242-18	Rectal swab	vanB
**37**	620-18	Rectal swab	vanB
**38**	684-18	Rectal swab	vanB
**39**	742-18	Rectal swab	vanB
**40**	764-18	Rectal swab	vanB
**41**	804-18	Rectal swab	vanB
**42**	1111-18	Rectal swab	vanB
**43**	1112-18	Rectal swab	vanB
**44**	1124-18	Rectal swab	vanB
**45**	1287-18	Rectal swab	vanB
**46**	1289-18	Rectal swab	vanB
**47**	1403-18	Rectal swab	vanB
**48**	1451-18	Rectal swab	vanB
**49**	1472-18	Rectal swab	vanB
**50**	1553-18	Rectal swab	vanB
**51**	1590-18	Rectal swab	vanB
**52**	1786-18	Rectal swab	vanB
**53**	1876-18	Rectal swab	vanB

**54**	1887-18	Rectal swab	vanB
**55**	1905-18	Rectal swab	vanB
**56**	1954-18	Rectal swab	vanB
**57**	2015-18	Rectal swab	vanB
**58**	2269-18	Rectal swab	vanB
**59**	1376-18	Rectal swab	vanB
**60**	2422-18	Rectal swab	vanB
**61**	3305-18	Blood culture	vanB
**62**	427-18	Rectal swab	vanB
**63**	428-18	Rectal swab	vanB
**64**	581-18	Rectal swab	vanB
**65**	3495-18	Rectal swab	vanB
**66**	2335-18	Rectal swab	vanB
**67**	2477-18	Blood culture	vanB
**68**	2905-18	Blood culture	vanB
**69**	3000-18	Blood culture	vanB
**70**	2063-19	Blood culture	vanB
**71**	2098-19	Blood culture	vanB
**72**	2168-19	Rectal swab	vanB
**73**	2608-19	Blood culture	vanB
**74**	3267-19	Rectal swab	vanB
**75**	2830-19	Rectal swab	vanB
**76**	3212-19	Blood culture	vanB
**77**	3217-19	Rectal swab	vanB
**78**	3388-19	Blood culture	vanB
**79**	3550-19	Rectal swab	vanB
**80**	3593-19	Blood culture	vanB
**81**	437-19	Rectal swab	vanB
**82**	655-19	Blood culture	vanB
**83**	241-19	Blood culture	vanB
**84**	458-19	Rectal swab	vanB
**85**	504-19	Rectal swab	vanB
**86**	640-19	Rectal swab	vanB
**87**	60-18	Blood culture	Susceptible
**88**	144-18	Blood culture	Susceptible
**89**	299-18	Blood culture	Susceptible
**90**	314-18	Blood culture	Susceptible
**91**	369-18	Blood culture	Susceptible
**92**	415-18	Blood culture	Susceptible
**93**	432-18	Blood culture	Susceptible
**94**	468-18	Blood culture	Susceptible
**95**	613-18	Blood culture	Susceptible
**96**	615-18	Blood culture	Susceptible
**97**	698-18	Blood culture	Susceptible
**98**	825-18	Blood culture	Susceptible
**99**	871-18	Blood culture	Susceptible
**100**	884-18	Blood culture	Susceptible
**101**	908-18	Blood culture	Susceptible
**102**	989-18	Blood culture	Susceptible
**103**	992-18	Blood culture	Susceptible
**104**	1002-18	Blood culture	Susceptible
**105**	1039-18	Blood culture	Susceptible
**106**	1053-18	Blood culture	Susceptible
**107**	1060-18	Blood culture	Susceptible
**108**	1116-18	Blood culture	Susceptible
**109**	1147-18	Blood culture	Susceptible
**110**	1162-18	Blood culture	Susceptible

**111**	1170-18	Blood culture	Susceptible
**112**	1293-18	Blood culture	Susceptible
**113**	1337-18	Blood culture	Susceptible
**114**	1401-18	Blood culture	Susceptible
**115**	1426-18	Blood culture	Susceptible
**116**	1498-18	Blood culture	Susceptible
**117**	1545-18	Blood culture	Susceptible
**118**	1615-18	Blood culture	Susceptible
**119**	1641-18	Blood culture	Susceptible
**120**	1717-18	Blood culture	Susceptible
**121**	1771-18	Blood culture	Susceptible
**122**	1791-18	Blood culture	Susceptible
**123**	2061-18	Blood culture	Susceptible
**124**	2095-18	Blood culture	Susceptible
**125**	2287-18	Blood culture	Susceptible
**126**	2439-18	Blood culture	Susceptible
**127**	3277-18	Blood culture	Susceptible
**128**	3312-18	Blood culture	Susceptible
**129**	37-19	Blood culture	Susceptible
**130**	131-19	Blood culture	Susceptible
**131**	310-19	Blood culture	Susceptible
**132**	328-19	Blood culture	Susceptible
**133**	345-19	Blood culture	Susceptible
**134**	368-19	Blood culture	Susceptible
**135**	378-19	Blood culture	Susceptible
**136**	390-19	Blood culture	Susceptible
**137**	395-19	Blood culture	Susceptible
**138**	430-19	Blood culture	Susceptible
**139**	460-19	Blood culture	Susceptible
**140**	464-19	Blood culture	Susceptible
**141**	552-19	Blood culture	Susceptible
**142**	559-19	Blood culture	Susceptible
**143**	575-19	Blood culture	Susceptible
**144**	595-19	Blood culture	Susceptible
**145**	598-19	Blood culture	Susceptible
**146**	604-19	Blood culture	Susceptible
**147**	676-19	Blood culture	Susceptible
**148**	950-19	Blood culture	Susceptible
**149**	965-19	Blood culture	Susceptible
**150**	994-19	Blood culture	Susceptible
**151**	1021-19	Blood culture	Susceptible
**152**	1033-19	Blood culture	Susceptible
**153**	1039-19	Blood culture	Susceptible
**154**	1241-19	Blood culture	Susceptible
**155**	1366-19	Blood culture	Susceptible
**156**	1468-19	Blood culture	Susceptible
**157**	1554-19	Blood culture	Susceptible
**158**	1590-19	Blood culture	Susceptible
**159**	1593-19	Blood culture	Susceptible
**160**	1625-19	Blood culture	Susceptible
**161**	1684-19	Blood culture	Susceptible
**162**	1703-19	Blood culture	Susceptible
**163**	1721-19	Blood culture	Susceptible
**164**	19152313	Rectal swab	Susceptible
**165**	19152964	Rectal swab	Susceptible
**166**	19152967	Rectal swab	Susceptible
**167**	19154177	Rectal swab	Susceptible

**168**	19154199	Rectal swab	Susceptible
**169**	19154200	Rectal swab	Susceptible
**170**	19154757	Rectal swab	Susceptible
**171**	19156397	Rectal swab	Susceptible
**172**	19156441	Rectal swab	Susceptible
**173**	19156448	Rectal swab	Susceptible
**174**	19157126	Rectal swab	Susceptible
**175**	19159082	Rectal swab	Susceptible
**176**	19159630	Rectal swab	Susceptible
**177**	19159642	Rectal swab	Susceptible
**178**	19159645	Rectal swab	Susceptible

Table S2. *E. faecium* isolates included in the repeatability assay. Their Pearson correlation coefficient for inter-days (biological) repeatability is shown.

**Samples**	**P Pearson**
"Enterococcus_faecium_S_1441"	0,856796493
"Enterococcus_faecium_S_41518"	0,968867739
"Enterococcus_faecium_S_82518"	0,988280503
"Enterococcus_faecium_S_96519"	0,968823737
"Enterococcus_faecium_S_98918"	0,965538082
"Enterococcus_faecium_S_100218"	0,989609441
"Enterococcus_faecium_S_124119"	0,945246999
"Enterococcus_faecium_S_171718"	0,888020473
"Enterococcus_faecium_S_327718"	0,969714629
"Enterococcus_faecium_S_19159642"	0,765205711
"Enterococcus_faecium_VanA_217"	0,988879506
"Enterococcus_faecium_VanA_2690"	0,990673724
"Enterococcus_faecium_VanA_2799"	0,984994445
"Enterococcus_faecium_VanA_2800"	0,990077871
"Enterococcus_faecium_VanA_3024"	0,953617436
"Enterococcus_faecium_VanB_2063"	0,908970353
"Enterococcus_faecium_VanB_2168"	0,91930336
"Enterococcus_faecium_VanB_3212"	0,966220338
"Enterococcus_faecium_VanB_3217"	0,908212247
"Enterococcus_faecium_VanB_3388"	0,898020244

Table S3. Coefficient of variation of the intensity means per level, normalization after (pTIC) and before (TICp) finding peaks and antibiotic susceptibility.

	**Intra-spots**			**Inter-Spots**			**Inter-Days**		
	**Not Normalized**	**TICp**	**pTIC**	**Not Normalized**	**TICp**	**pTIC**	**Not Normalized**	**TICp**	**pTIC**
TOTAL	15.35442	8.460269	7.999143	29.29323	20.88475	19.90829	31.24830	20.65608	19.29570
SUSCEPTIBLE	17.66087	9.102237	8.446666	23.68497	17.3779	16.92136	33.55983	22.47998	20.9593
RESISTANT	12.07580	7.538624	7.356173	33.96677	23.80711	22.39739	28.93677	18.83218	17.63204

Table S4. Comparison of the p-values between average coefficient of variation of intensity means for peaks from 2000 to 3000 *m*/*z* and 3000 to 9000 *m*/*z* range.

	Not normalized	TICp	pTIC
**Days**	0.9549	2.68E-06	0.000934
**Spots**	0.5663	0.000528	0.001684
**Shoots**	3.76E-05	2.77E-05	2.73E-05

Table S5. p-values for arithmetic and post-alignment means at spot- and day level with and without normalization.

p-Value	Not Normalized	TICp
**Inter-Spots Mean**	0.8366	0.09755
**Inter-Days Mean**	0.9298	0.1283

Table S6. Accuracy and F1-factor of the internal validation for the classification of Vancomycin-resistant *E. faecium* (VRE) and Vancomycin-susceptible *E. faecium* (VSE) strains as metric for algorithms scores. Data obtained with k-fold cross validation method where k=10. Scaled data.

	Full spectrum-TIC		Peaks-TIC		TIC-Peaks	
	**Accuracy**	**F1 Score**	**Accuracy**	**F1 Score**	**Accuracy**	**F1 Score**
**Partial Least Squares (4 Components)**	77.53%	75.31%	72.47%	70.66%	76.4%	74.7%
**Support Vector Machine Linear SVC (liblinear)**	**80.9**%	80.46%	70.22%	70.72%	76.97%	75.45%
**Random Forest**	79.21%	77.85%	75.84%	74.56%	72.47%	70.3%

Table S7. Accuracy of internal validation for the classification of vanA and vanB Vancomycin-resistant *E. faecium* (VRE) strains as metric for algorithms scores. Data obtained with k-fold cross validation method where k=10. Scaled data.

	Full spectrum-TIC	Peaks-TIC	TIC-Peaks
**Partial Least Squares (4 Components)**	83.72%	83.72%	**86.65%**
**Support Vector Machine - LinearSVC (liblinear)**	76.74%	80.23	73.26
**Random Forest**	79.07%	80.23%	79.07%

Figure S1. Area Under the Curve (AUC) values for the two potential biomarkers found for A) Vancomycin-resistant *E. faecium* (VRE) isolates -5095.01 *m/z*- and B) for vanA VRE isolates -6891.33 *m/z*-.

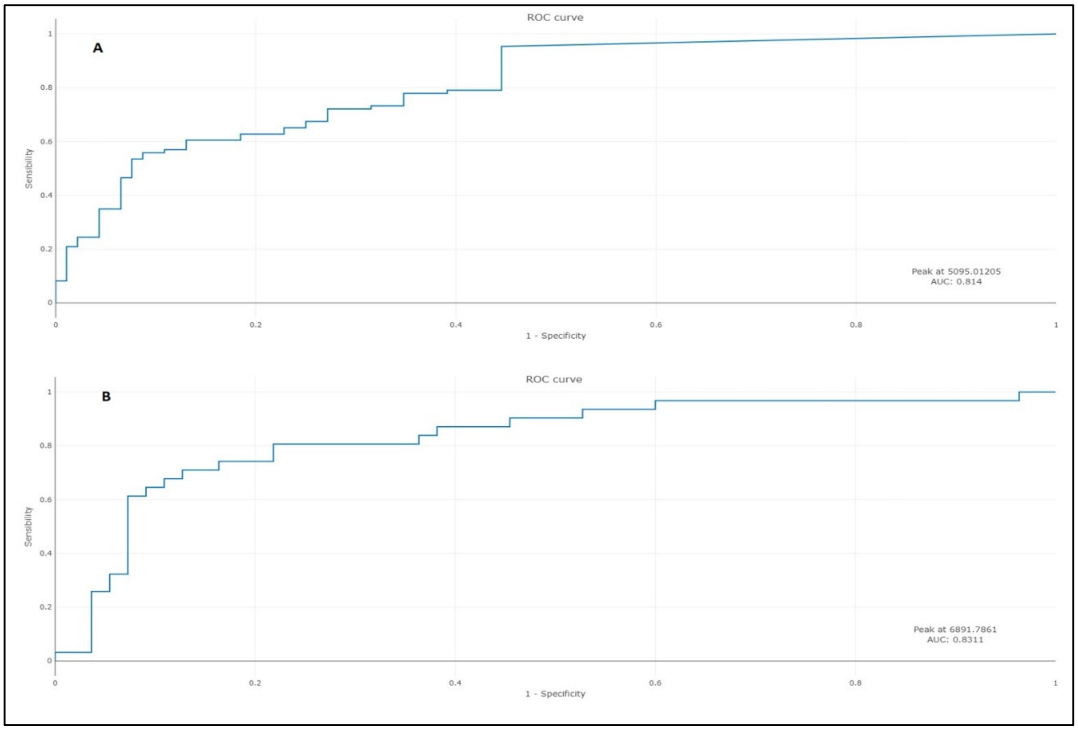

Figure S2. Visual discrimination of protein spectra from Vancomycin-resistant *E. faecium* (VRE) –in red- and Vancomycin-susceptible *E. faecium* (VSE) –green-according to the presence/absence of the peak at 5095.01 *m/z* (A). Differentiation of van A –lblue- and vanB VRE isolates –purple- was based on the intensity of the peak at 6891.33 *m/z* (B).

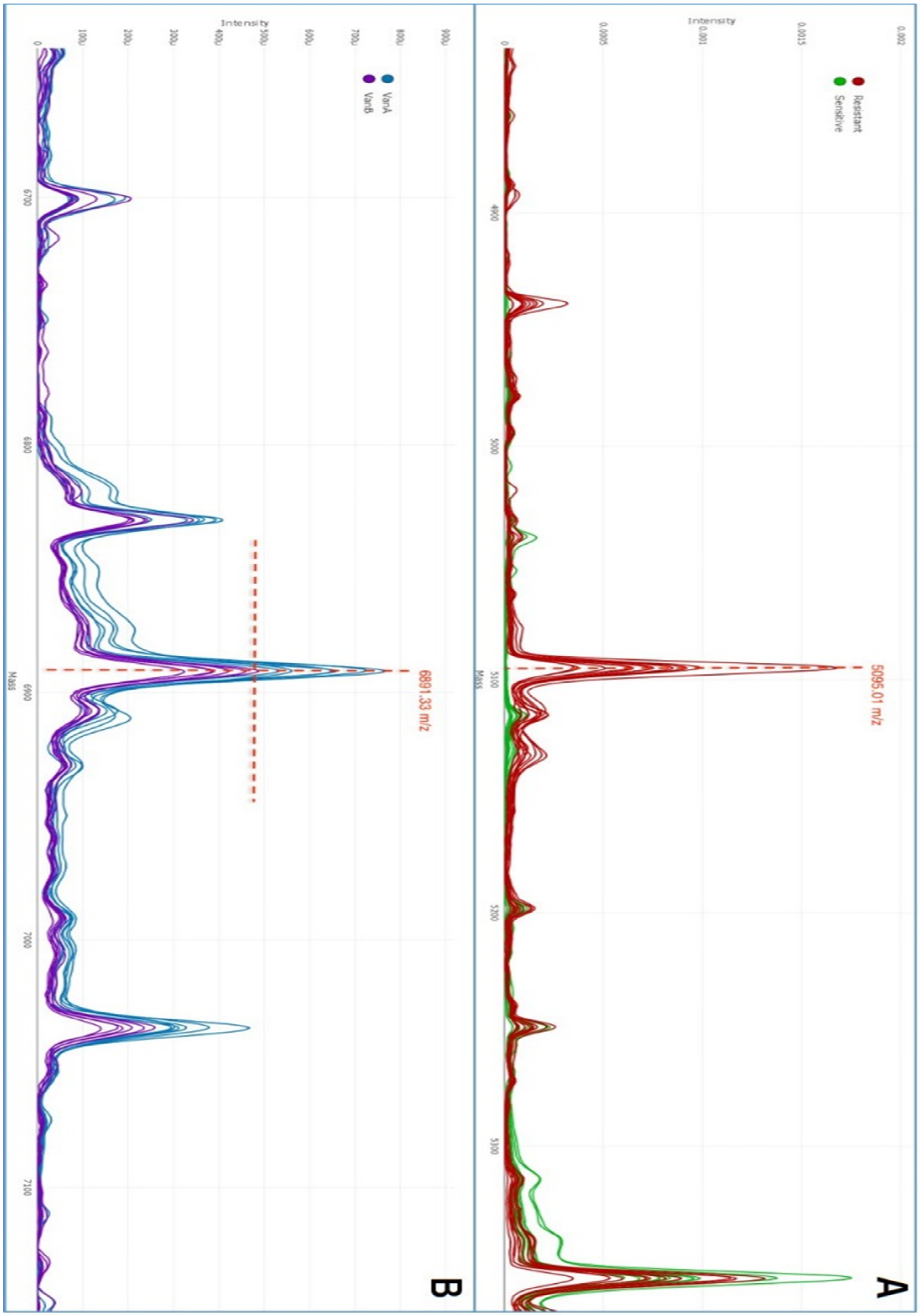

Supplement: Supplementary file 1 [file diagnostics-12-00328-s001.zip › diagnostics-1555210-supplementary.pdf]
